# Isoleucine Enhances Plant Resistance Against *Botrytis cinerea* via Jasmonate Signaling Pathway

**DOI:** 10.3389/fpls.2021.628328

**Published:** 2021-08-19

**Authors:** Yuwen Li, Suhua Li, Ran Du, Jiaojiao Wang, Haiou Li, Daoxin Xie, Jianbin Yan

**Affiliations:** ^1^Tsinghua-Peking Center for Life Science, and MOE Key Laboratory of Bioinformatics, School of Life Sciences, Tsinghua University, Beijing, China; ^2^Shenzhen Branch, Guangdong Laboratory for Lingnan Modern Agriculture, Genome Analysis Laboratory of the Ministry of Agriculture and Rural Affairs, Agricultural Genomics Institute at Shenzhen, Chinese Academy of Agricultural Sciences, Shenzhen, China; ^3^Shenzhen Key Laboratory of Agricultural Synthetic Biology, Agricultural Genomics Institute at Shenzhen, Chinese Academy of Agricultural Sciences, Shenzhen, China; ^4^Hunan Provincial Key Laboratory of Phytohormones and Growth Development, College of Bioscience and Biotechnology, Hunan Agricultural University, Changsha, China

**Keywords:** isoleucine, *Botrytis cinerea*, JA-Ile, COI1, JAR1

## Abstract

Amino acids are the building blocks of biomacromolecules in organisms, among which isoleucine (Ile) is the precursor of JA-Ile, an active molecule of phytohormone jasmonate (JA). JA is essential for diverse plant defense responses against biotic and abiotic stresses. *Botrytis cinerea* is a necrotrophic nutritional fungal pathogen that causes the second most severe plant fungal disease worldwide and infects more than 200 kinds of monocot and dicot plant species. In this study, we demonstrated that Ile application enhances plant resistance against *B. cinerea* in *Arabidopsis*, which is dependent on the JA receptor COI1 and the jasmonic acid-amido synthetase JAR1. The mutant *lib* with higher Ile content in leaves exhibits enhanced resistance to *B. cinerea* infection. Furthermore, we found that the exogenous Ile application moderately enhanced plant resistance to *B. cinerea* in various horticultural plant species, including lettuce, rose, and strawberry, suggesting a practical and effective strategy to control *B. cinerea* disease in agriculture. These results together showed that the increase of Ile could positively regulate the resistance of various plants to *B. cinerea* by enhancing JA signaling, which would offer potential applications for crop protection.

## Introduction

*Botrytis cinerea* is a necrotrophic nutritional fungal pathogen that infects more than 200 kinds of monocot and dicot plants and subsequently causes gray mold disease, which is the second most common plant fungal disease worldwide (Williamson et al., 2007; Dean et al., 2012; AbuQamar et al., 2017). *B. cinerea* infection may occur from the seedling stage to fruit ripening stage and even during the storage and transport in the retail chain (Dean et al., 2012). Global costs are greater than €1 billion annually for *B. cinerea* control, which includes agronomic and horticultural practices, fungicides, biological control, and postharvest treatments (Dean et al., 2012; Petrasch et al., 2019). However, increasing fungicide resistance is a severe challenge to fungicide applications and the agricultural management practice for *B. cinerea* (Kretschmer et al., 2009; Rupp et al., 2016). In addition, *Bacillus subtilis*-based biological control is also limited by insufficient applicability in the field and high costs (Petrasch et al., 2019). Therefore, safer, pollution-free, and affordable strategies for controlling *B. cinerea* are worthy of discovery.

Amino acids are the building blocks of biomacromolecules in organisms, and they are also used as water-soluble fertilizers to promote plant growth and improve crop quality (Wang et al., 2014; Aghaei et al., 2019; Rahmani Samani et al., 2019). For instance, the application of amino acids promotes root growth and increases leaf area, chlorophyll content, and dry weight in rapeseed (Wang et al., 2014). Some amino acids are precursor components for the synthesis of various secondary defensive metabolites. For instance, aliphatic amino acids (i.e., alanine, leucine, isoleucine [Ile], methionine, and valine) and aromatic amino acids (i.e., phenylalanine, tryptophan, and tyrosine) involve in the formation of aliphatic, aromatic, and indolic glucosinolates (Halkier and Gershenzon, 2006; Albinsky et al., 2010). The hydrolysis products of glucosinolates serve as defense compounds against herbivores and pathogens and act as cancer-preventing agents in humans (Halkier and Gershenzon, 2006). In addition, arginine and citrulline activate the nitric oxide (NO) cycle to restore the susceptibility of brown planthoppers to the insecticide imidacloprid (Elzaki et al., 2020). Glutamate activates the salicylic acid (SA) pathway to trigger the blast resistance in rice (Kadotani et al., 2016). Despite the apparent relevance between amino acid metabolism and disease resistance, the molecular basis for amino acid and plant resistance to *B. cinerea* has not been elucidated to date.

Isoleucine is synthesized from threonine under the catalysis by threonine deaminase (TD), encoded by the *L-O-METHYLTHREONINE-RESISTANT 1* (*OMR1*) gene in *Arabidopsis* (Kang et al., 2006; Binder, 2010; Yu et al., 2013). A previous study reported that the low Ile biosynthesis mutant (*lib*) with a T-DNA insertion in the *OMR1* gene exhibits reduced Ile levels in roots and defects in cell proliferation and expansion during root development (Yu et al., 2013). Furthermore, Ile also conjugates with jasmonic acid under the catalysis by jasmonic acid-amido synthetase JAR1 to form JA-Ile, a bioactive molecule of jasmonates (JAs) (Staswick et al., 2002; Staswick and Tiryaki, 2004; Fonseca et al., 2009; Yan et al., 2016). Subsequently, JA-Ile binds to its receptor COI1 (Xie et al., 1998; Xu et al., 2002; Thines et al., 2007; Yan et al., 2009, 2013) to mediate plant resistance against *B. cinerea* infection (Thaler et al., 2004; Abuqamar et al., 2008; Song et al., 2013). In the JA biosynthetic pathway, α-linolenic acid is catalyzed and converted into jasmonic acid through a series of biosynthetic enzymes, including allene oxide synthase (AOS) and 12-oxophytodienoate reductase 3 (OPR3) (Wasternack and Hause, 2013).

In this study, we screened 20 proteinogenic amino acids and found that exogenous application of Ile enhanced plant resistance to *B. cinerea* in *Arabidopsis*. Our results showed that the Ile-enhanced resistance is modulated *via* JA signaling through COI1 and JAR1. Notably, the application of Ile on horticultural plant species, such as lettuce, white rose, red rose, and strawberry, moderately enhanced resistance to *B. cinerea*, suggesting that increasing Ile levels to improve disease resistance has broad applicability and great potential in agriculture.

## Results

### Exogenous Application of Isoleucine Enhances Plant Resistance to *B. cinerea*

**FIGURE 1:**
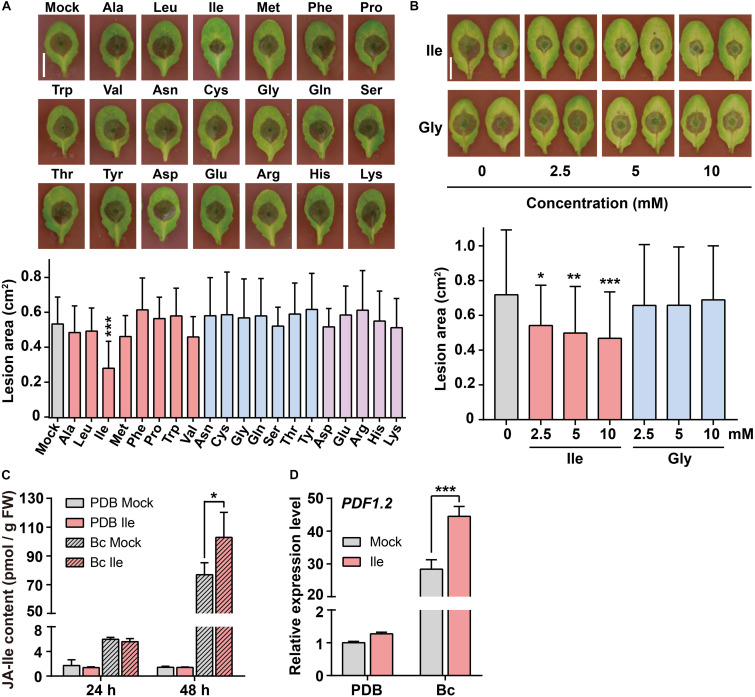
Application of isoleucine (Ile) enhances plant resistance to *Botrytis cinerea via* elevating JA-Ile (jasmonoyl-isoleucine) level. **(A)** Representative phenotype and lesion area of wild-type (WT) leaves pretreated with different amino acids after *B. cinerea* infection for 3 days. Five-week-old WT plants were pretreated with 10 mM amino acids for 2 days before *B. cinerea* inoculation, including non-polar amino acids (i.e., Ala, Leu, Ile, Met, Phe, Pro, Trp, and Val), polar amino acids (i.e., Asn, Cys, Gly, Gln, Ser, Thr, and Tyr), acidic amino acids (i.e., Asp and Glu), and alkaline amino acids (i.e., Arg, His, and Lys). 0.05% Tween 20 solution (Mock) was used as a control. Scale: 1 cm. Data are means ± SD (*n* = 28–47 leaves). **(B)** Representative phenotype and lesion area of WT leaves after *B. cinerea* infection for 3 days pretreated with different concentrations of Ile or Gly. Five-week-old plants were pretreated with 0, 2.5, 5, and 10 mM Ile or Gly for 2 days. 0.05% Tween 20 solution (0 mM) was used as control. Scale: 1 cm. Data are means ± SD (*n* = 24–43 leaves). **(C,D)** JA-Ile concentration **(C)** and transcript level of the defensive gene *PDF1.2* **(D)** in WT leaves after indicated treatments. Four-week-old WT plants were pretreated with 0.05% Tween 20 solution (Mock) or 10 mM Ile for 2 days, and then leaves were sprayed with Potato Dextrose Broth (PDB) or *B. cinerea* spores suspension (Bc) for 24 or 48 h. Then, the 7th–9th rosette leaves were collected for quantification of JA-Ile contents, and *PDF.1.2* transcript level (48 h). Data are means ± SD. In panel **(C)**, *n* = 4 samples. In panel **(D)**, *n* = 3 samples, each sample contains three leaves; *ACTIN8* is used as the internal control. Statistical significances were calculated *via* Student’s *t*-test Mock and amino acid treatment (*p < 0.05; **p < 0.01; ***p < 0.001).

We sprayed 20 amino acids on *Arabidopsis* wild-type (WT) plants Col-0 for 2 days and subsequently inoculated *B. cinerea* spores on the leaves to examine the effects of amino acids on plant defense against *B. cinerea* infection. The lesion area on leaves caused by *B. cinerea* infection was measured on the third day after inoculation. Ile treatment (10 mM) significantly reduced the leaf lesion size compared with the control (Mock) treatment (Figure 1A). Applications of Ala, Leu, Val, and Met also slightly reduced lesion area from 7 to 13% (Figure 1A). In summary, only exogenous application of Ile obviously enhanced plant resistance to *B. cinerea*, while other amino acids exhibited no significant difference compared with the Mock treatment (Figure 1A).

We further examined the effects of different concentrations of Ile on plant resistance to *B. cinerea* infection. As shown in Figure 1B, the lesion size on WT leaves was reduced ∼24% when plants were pretreated with 2.5 mM Ile, ∼30% when pretreated with 5 mM Ile, and ∼38% when pretreated with 10 mM Ile. The lesion area on WT leaves pretreated with glycine (Gly) at various concentrations has no significant difference compared with solvent treatment (Figure 1B). These results suggested that the exogenous application of Ile enhanced plant defense against *B. cinerea* in a concentration-dependent manner.

As Ile is the precursor of JA-Ile, an important defensive signal in response to *B. cinerea* infection (Thaler et al., 2004; Abuqamar et al., 2008; Song et al., 2013), we further measured JA-Ile accumulation using liquid chromatography–tandem mass spectrometry (LC-MS/MS). LC-MS/MS analysis showed that JA-Ile concentration in Ile-pretreated WT leaves was higher than control WT leaves after *B. cinerea* infection for 48 h (Figure 1C). Consistently, the induction of defensive gene *PDF1.2* (*PLANT-DEFENSIN1.2*) was also stronger in Ile-pretreated WT leaves compared with control WT leaves at 48 h upon *B. cinerea* infection (Figure 1D). Collectively, Ile-enhanced resistance may be caused by the higher accumulation of JA-Ile.

### The Increase of Endogenous Ile Level Enhances Plant Resistance to *B. cinerea*

**FIGURE 2:**
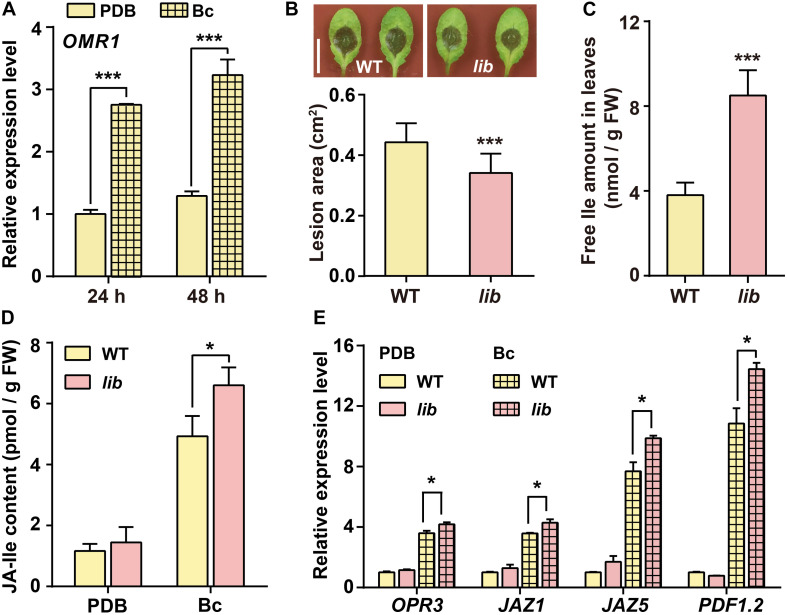
*lib* mutant shows enhanced plant resistance to *B. cinerea*. **(A)** Transcript level of Ile biosynthetic gene *OMR1* in WT leaves. Four-week-old WT plants were sprayed with Potato Dextrose Broth (PDB) or *B. cinerea* spores suspension (Bc) for 24 and 48 h. Data are means ± SD (*n* = 3 samples, each sample contains three leaves). *ACTIN8* was used as the internal control. **(B)** Representative phenotype and lesion area of WT and *lib* leaves after *B. cinerea* infection for 2 days. Data are means ± SD (*n* = 61–69 leaves). **(C)** Free Ile amount in the leaves of WT and *lib* seedlings. Five-day-old seedlings were transferred to vertical MS medium (1.2% agar) plates for 10 days, and then leaves were collected for Ile measurement. Data are means ± SD (*n* = 7–10). **(D,E)** JA-Ile content **(D)** and transcript level of JA biosynthetic gene *OPR3*, JA-responsive genes *JAZ1* and *JAZ5*, and defensive gene *PDF1.2* **(E)** in the leaves after indicated treatments. Three-week-old WT and *lib* plants were sprayed with PDB or *B. cinerea* spores suspension (Bc) for 24 h. Then, rosette leaves were collected for quantification of JA-Ile contents and transcript level. Data are means ± SD. In panel **(D)** *n* = 3–7 samples. In panel **(E)** *n* = 3 samples, each sample contains three leaves; *ACTIN8* is used as the internal control. Asterisks indicate significant differences (Student’s *t*-test, *p < 0.05; ***p < 0.001).

The conversion of threonine to 2-oxobutanoate, catalyzed by OMR1 enzyme, is the first and committed step toward Ile biosynthesis in *Arabidopsis* (Kang et al., 2006; Binder, 2010; Yu et al., 2013). Our results showed that *OMR1* gene transcription level was highly induced in *B. cinerea*-infected leaves (Figure 2A). A previous study reported that *lib* mutant, a T-DNA insertion in the last exon of *OMR1* gene causing a 13-amino-acid truncation at C-terminal region of OMR1 protein, showed reduced Ile levels in roots (Yu et al., 2013). Hence, we further analyzed the disease resistance response of *lib* mutant to examine the effect of endogenous Ile levels on plant resistance to *B. cinerea*. Unexpectedly, the lesion area of *lib* leaves was ∼23% reduced compared with that of WT (Figure 2B). To clarify whether the enhanced resistance to *B. cinerea* of *lib* mutant is linked to free amino acid contents, we further detected the amount of free amino acids in the leaves. As shown in Supplementary Table 1 and Figure 2C, the Ile level was significantly increased in *lib* leaves by approximately 1-fold higher than WT. In addition, we also found that Gly content in *lib* leaves was higher than that in WT leaves (Supplementary Table 1). However, external Gly treatment on WT leaves failed to enhance plant resistance to *B. cinerea* (Figure 1B), which further suggested that the enhanced resistance of *lib* mutant is linked to the higher level of Ile but not Gly. Based on exogenous and endogenous results, we speculated that Ile plays a positive role in plant resistance to *B. cinerea*.

In line with the enhanced resistance of *lib* mutant, the transcript level of defensive gene *PDF1.2* in *lib* leaves was higher than that in WT leaves followed by *B. cinerea* infection for 48 h, whereas *OPR3* level in *lib* leaves was comparable with that in WT (Supplementary Figure 1). Transcriptomic analysis showed that JA-responsive genes are overrepresented around 16 h after *B. cinerea* infection, suggesting prior JA biosynthesis (Windram et al., 2012). Therefore, we further investigated whether the enhanced disease resistance to *B. cinerea* of *lib* mutant was related to JA-Ile biosynthesis and signaling at an earlier stage of *B. cinerea* infection.

First, after *B. cinerea* infection, WT and *lib* leaves were collected for JA-Ile measurement using LC-MS/MS. The LC-MS/MS analysis showed that the JA-Ile content was significantly increased in *lib* leaves on *B. cinerea* infection for 24 h. JA-Ile increased to ∼6.6 pmol/g fresh weight (FW) in *lib* leaves, whereas it increased only to ∼4.9 pmol/g FW in WT leaves (Figure 2D). Consistent with higher JA-Ile contents and stronger disease resistance in *lib* plants following *B. cinerea* infection, the transcript levels of the JA biosynthetic gene *OPR3*, JA-responsive genes *JAZ1* and *JAZ5*, and defensive gene *PDF1.2* were significantly induced in *lib* mutant, which was higher than that in WT plants (Figure 2E). These data demonstrated that an increase of endogenous Ile level could enhance plant disease resistance *via* triggering JA-Ile biosynthesis and signaling on *B. cinerea* infection.

### Ile-Enhanced Resistance to *B. cinerea* Depends on Elevated JA-Ile Biosynthesis and Perception

**FIGURE 3:**
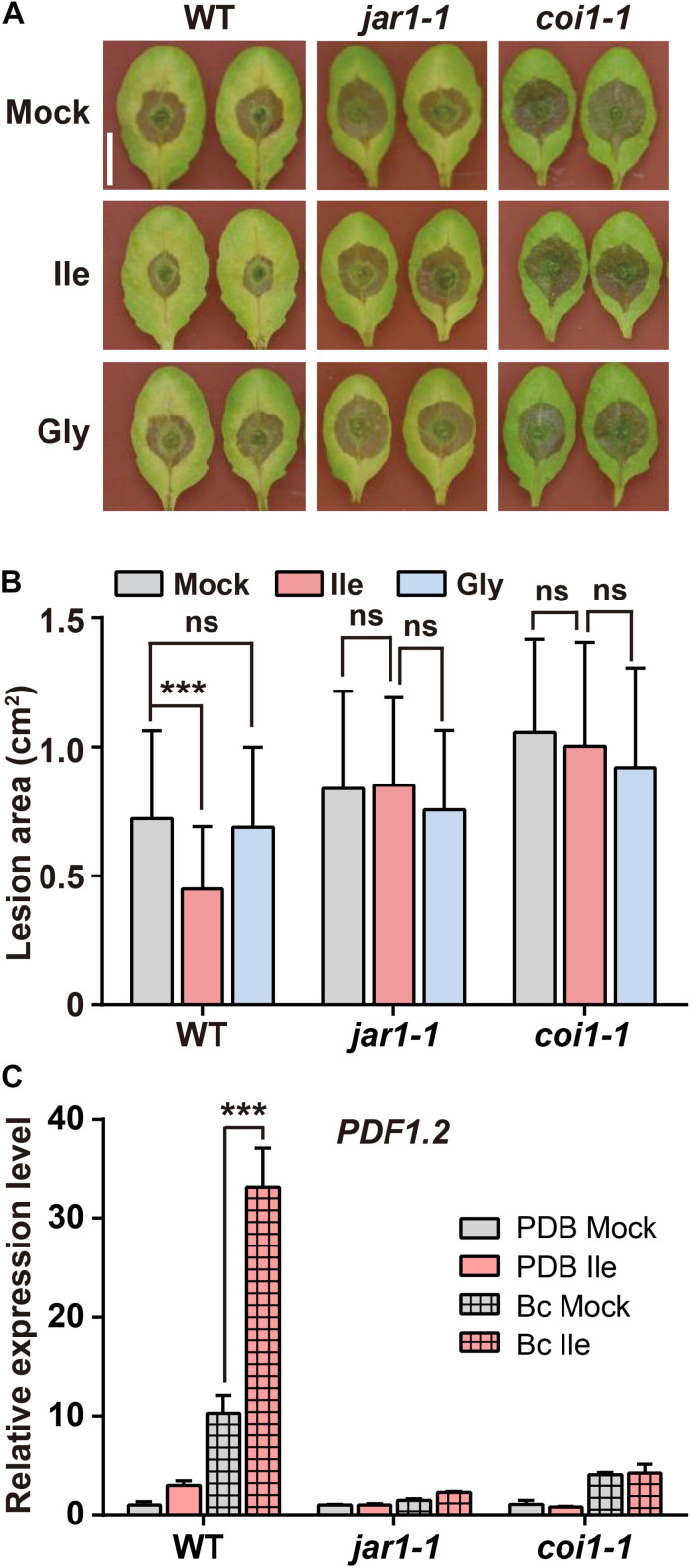
Ile enhances plant resistance to *B. cinerea* depending on JAR1 and COI1. **(A,B)** Representative phenotype **(A)** and lesion area **(B)** of WT, *jar1-1*, and *coi1-1* leaves after *B. cinerea* infection for 3 days. Five-week-old plants were pretreated with 0.05% Tween 20 solution (Mock), 10 mM Ile, or 10 mM Gly for 2 days before *B. cinerea* inoculation. Scale: 1 cm. Data are means ± SD (*n* = 32–51 leaves). **(C)** Transcript level of defensive gene *PDF1.2* in the leaves of WT, *jar1-1*, and *coi1-1* plants. Four-week-old plants were pretreated with 0.05% Tween 20 solution (Mock) or 10 mM Ile for 2 days. The 7th–9th rosette leaves were detached and inoculated with PDB or *B. cinerea* spores suspension (Bc) for 48 h, and then collected for quantification of *PDF1.2* transcript level. Data are means ± SD (*n* = 3 samples, each sample contains three leaves). *ACTIN8* is used as the internal control. Statistical significances were calculated *via* Student’s *t*-test (***p < 0.001, “ns” *p* > 0.05).

The biosynthesis and perception of JA-Ile are required for plant defense against *B. cinerea* (Thaler et al., 2004; Abuqamar et al., 2008; Song et al., 2013). Hence, we investigated whether the Ile-enhanced resistance to *B. cinerea* after Ile application depends on elevated JA-Ile biosynthesis and signaling using *jar1-1* (Staswick et al., 2002) and *coi1-1* (Xie et al., 1998) mutants. The results showed that Ile application significantly reduced the size of lesion in WT leaves, but it failed to reduce the size of lesion in *jar1-1* and *coi1-1* mutants (Figures 3A,B). However, treatments with Gly had no significant effect on the lesion size of WT, *coi1-1*, and *jar1-1* mutants (Figures 3A,B). In line with these results, the transcript level of defensive gene *PDF1.2* in Ile-pretreated WT leaves on *B. cinerea* infection was higher than that in Mock-pretreated leaves; however, the acceleration of Ile on *PDF1.2* expression on *B. cinerea* infection was not observed in *jar1-1* and *coi1-1* mutants (Figure 3C). These results demonstrated that the enhanced resistance to *B. cinerea* triggered by Ile depends on the elevated JA-Ile biosynthesis and perception through JAR1 and COI1.

### High Endogenous Ile Level Enhances JA Responses to Wounding and MeJA Treatment

In addition to *B. cinerea* infection, JAs play essential roles in regulating herbivore defenses, wounding responses, fertility, anthocyanin accumulation, UV irradiation, and drought stress (Creelman and Mullet, 1995; Xie et al., 1998; Farmer et al., 2003; Qi et al., 2011; Seo et al., 2011; Wathugala et al., 2012; Yan et al., 2018). Therefore, we performed wounding treatment and MeJA treatment to further investigate whether the increase of Ile level affects other JA responses in addition to *B. cinerea* infection.

**FIGURE 4:**
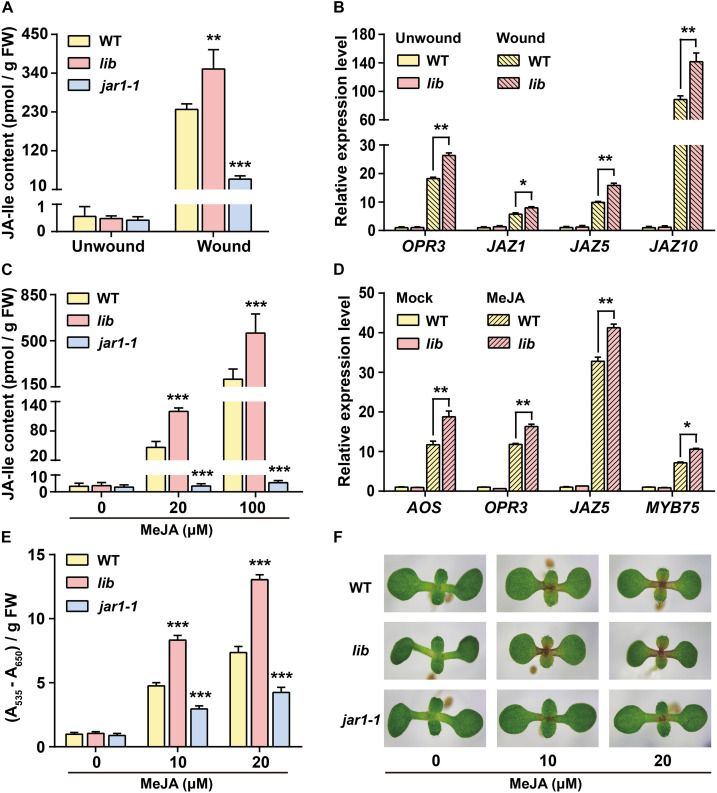
Jasmonate responses are enhanced in *lib* mutants after wounding and MeJA treatment. **(A)** JA-Ile content in unwounded and wounded leaves of 4-week-old WT, *lib*, and *jar1-1* seedlings. Data are means ± SD (*n* = 5–6 samples). **(B)** Transcript level of JA biosynthetic gene *OPR3* and JA-responsive genes *JAZ1, JAZ5*, and *JAZ10* in unwounded and wounded leaves of 4-week-old WT and *lib* plants. *ACTIN8* was used as the internal control. Data are means ± SD (*n* = 3 samples). **(C)** JA-Ile content in 14-day-old WT, *lib*, and *jar1-1* seedlings treated with 0, 20, and 100 μM MeJA for 1 h. Data are means ± SD (*n* = 5–6 samples). **(D)** Transcript level of the JA biosynthetic genes *AOS and OPR3* and the JA-responsive genes *JAZ5* and *MYB75* in 7-day-old WT and *lib* seedlings treated with solvent (Mock) or 50 μM MeJA for 1 h. *ACTIN8* was used as the internal control. Data are means ± SD (*n* = 2–3 samples, each sample contains 20 seedlings). **(E,F)** Anthocyanin contents **(E)** and representative phenotype **(F)** of WT, *lib*, and *jar1-1* seedlings grown on MS medium supplemented with 0, 10, and 20 μM MeJA for 8 days. Data are means ± SD (*n* = 3 samples). Asterisks indicate significant differences compared with WT (Student’s *t*-test, *p < 0.05; **p < 0.01; ***p < 0.001).

To do so, we first compared the wounding responses in *lib*, *jar1-1*, and WT plants. Rosette leaves of these plants were subjected to wounding treatment for 1 h and subsequently collected for the measurement of endogenous JA-Ile concentration using LC-MS/MS (liquid chromatography-tandem mass spectrometry). Consistent with previous studies (Suza and Staswick, 2008; Yan et al., 2016), the induction of JA-Ile level by wounding was impaired in *jar1-1* mutants compared with that in WT leaves (Figure 4A). JA-Ile accumulated more dramatically in the wounded leaves of *lib* mutant. JA-Ile levels increased up to ∼352 pmol/g FW in *lib*, ∼237 pmol/g FW in WT, and ∼40 pmol/g FW in the *jar1-1* mutant (Figure 4A). Consistent with the increased JA-Ile contents in *lib* mutants, the transcript level of JA-inducible genes in *lib* leaves, such as *OPR3*, *JAZ1*, *JAZ5*, *and JAZ10*, was significantly higher than that in WT leaves on wounding treatment (Figure 4B).

It has been demonstrated by isotope-feeding experiments that exogenous MeJA could be converted into JA and further conjugated with Ile into JA-Ile to activate JA signaling pathway (Tamogami et al., 2008). Therefore, we further examined whether increased endogenous Ile levels in *lib* leaves enhance its capacity for MeJA-induced JA-Ile biosynthesis. Here, 14-day-old WT, *lib*, and *jar1-1* seedlings were subjected to MeJA treatment for 1 h and then collected for endogenous JA-Ile measurement. On 20 μM MeJA treatment, JA-Ile levels were induced to ∼120 pmol/g FW in *lib*, to ∼46 pmol/g FW in WT, and ∼3.5 pmol/g FW in *jar1-1* plants (Figure 4C). Similarly, on 100 μM MeJA treatment, JA-Ile increased to ∼558 pmol/g FW in *lib* leaves, ∼208 pmol/g FW in WT, and ∼5.5 pmol/g FW in *jar1-1* leaves (Figure 4C). As a result, the induction of JA-Ile biosynthesis in *lib* leaves was significantly higher than that in WT leaves, suggesting that the high endogenous Ile contributes to more conjugation of Ile with JA (MeJA-derived) into JA-Ile. In addition, JA-Ile biosynthesis was impaired in *jar1-1* mutants on MeJA treatment. As in *jar1-1* mutants, the mutated JAR1 enzyme lost the ability to catalyze JA and Ile into JA-Ile, suggesting that the exogenous MeJA-induced JA-Ile accumulation is dependent on JAR1. Consistently, JA-responsive genes, such as *AOS*, *OPR3*, *MYB75*, *JAZ5*, *PDF1.2*, and *VSP1*, were obviously induced in *lib* mutants on MeJA treatment (Figure 4D and Supplementary Figures 2B–E).

We also compared MeJA-induced anthocyanin accumulation among WT, *lib*, and *jar1-1* seedlings. WT, *lib*, and *jar1-1* seedlings were grown on MS medium supplemented with 0, 10, and 20 μM MeJA for 8 days and collected for the measurement of anthocyanin contents as well as expression levels of the anthocyanin biosynthetic gene *GL3.* These data showed that the anthocyanin content and expression level of *GL3* in *lib* mutants were dramatically increased compared with those in WT leaves. In contrast, the accumulation of anthocyanin and the induction of *GL3* were impaired in the *jar1-1* mutant (Figures 4E,F and Supplementary Figure 2A). Taken together, our findings suggested that a higher Ile level in *lib* mutant improved its capacity to convert MeJA into JA-Ile and triggered JA signaling.

In summary, wound- and MeJA-triggered JA responses in *lib* mutant are significantly higher than WT, which demonstrated that the increase of Ile levels in *lib* leaves enhanced JA responses. These results provided a possibility that the increase of Ile level may improve plant resistance to other environmental stresses in addition to *B. cinerea* infection.

### Increased Endogenous Ile Level Has No Effect on Aboveground Growth of *Arabidopsis*

The *lib* mutant exhibited similar JA-Ile contents with WT plants under resting conditions (Figure 4). Next, the effect of the increase of Ile contents in *lib* mutants on plant growth and development was investigated. First, we observed the development of the aboveground parts of *lib* plants at various stages. As shown in Supplementary Figures 3A,B, FW of the aboveground parts of *lib* mutants had no obvious difference compared with WT at the 18–, 23–, 28–, and 33-day-old stages. In addition, the morphology of flower, the dehiscence of anther, the main inflorescence, and seeds development did not exhibit an obvious difference between WT and *lib* plants (Supplementary Figure 3A). The number of mature siliques and the seed production also did not exhibit a significant difference between *lib* and WT plants (Supplementary Figures 3C,D). These results suggested that a proper increase of endogenous Ile level had no obvious effect on plant growth, development, and seed production.

### Ile Application Broadly Improves Resistance to *B. cinerea* in Various Plants

*Botrytis cinerea* also infects various plants of economic interest, including lettuce, rose, and strawberries. Lettuce (*Lactuca sativa*, Compositae) is an annual and vitamin-rich leaf vegetable that can be damaged by *B. cinerea* and causes water-soaked, brownish-gray to brownish-orange symptoms (Shim et al., 2014). Rose (*Rosa hybrida L.*, Rosaceae) is one of the most ornamental plants and is used as a food as well as in traditional medicines in China. Fresh cut roses are generally transported from farmers to customers. During long-distance transportation, gray mold disease caused by *B. cinerea* leads to the most serious postharvest loss of rose production (Cao et al., 2019). Strawberry (*Fragaria* × *ananassa*, Rosaceae) is a popular fruit worldwide and is also threatened by *B. cinerea*, which infects all aerial parts of the plant, especially flowers and fruits, and results in a greater than 50% yield loss (Petrasch et al., 2019). *B. cinerea* infection of these plants causes billions of dollars in loss (Qi et al., 2018; Cao et al., 2019). Therefore, we next explored whether the enhanced resistance caused by the Ile application is of universal significance in these plant species.

**FIGURE 5:**
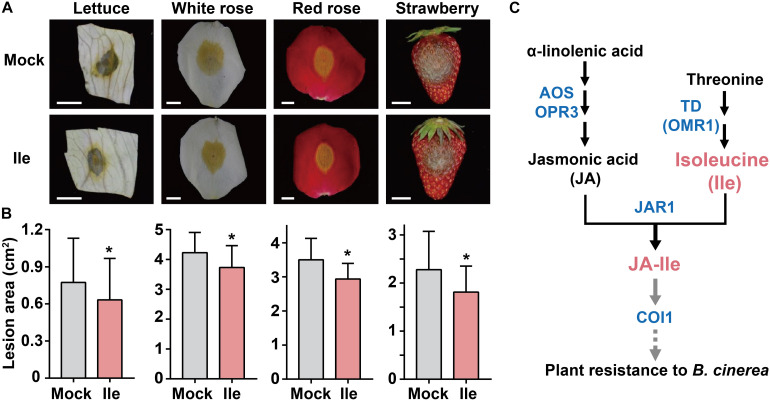
Application of Ile improves plant resistance to *B. cinerea* in horticultural plants. **(A,B)** Representative phenotype **(A)** and lesion area **(B)** of lettuce, roses, and strawberry after *B. cinerea* infection. 10 mM Ile and 0.05% Tween 20 solution (Mock) were respectively sprayed on lettuce leaves, flowers (white rose and red rose), and strawberries for 2 days before inoculation. Then, the detached indicated parts of plants were inoculated with *B. cinerea* spores suspension (1.0 × 10^6^ spores/ml), moisturized in 1% agar dishes, and incubated in the dark for indicated days. Lesion sizes were measured and analyzed after 2 days for white rose (*n* = 57–58), 3 days for lettuce (*n* = 54–57) and red rose (*n* = 24–26), and 4 days for strawberry (*n* = 32). Scale: 1 cm. Data are means ± SD. Asterisks indicate significant differences compared with Mock treatment (Student’s *t*-test, *p < 0.05). **(C)** Regulation model of *B. cinerea* resistance mediated by Ile through JA signaling. JA is synthesized from α-linolenic acid through serial key enzymes, such as Allene oxide synthase (AOS) and OPDA reductase 3 (OPR3). Threonine deaminase (OMR1) catalyzes the conversion of threonine into 2-oxobutanoate, which is the first and the committed step in Ile biosynthesis. JA-Ile is then synthesized by conjugating jasmonic acid (JA) with Ile *via* JA-amido synthetase JAR1. On *B. cinerea* infection, a high Ile level will contribute to stronger induction of JA-Ile biosynthesis and then lead to enhanced JA responses depending on JAR1 and COI1. Black arrows represent the schematic diagram of JA-Ile biosynthesis. Gray arrows represent that JA-Ile is recognized by receptor COI1, subsequentially activating signal transduction to defense against *B. cinerea* infection.

We applied 10 mM Ile to the leaves of lettuce, the flowers of white rose and red rose, and the fruits of strawberry for 2 days followed by inoculation with *B. cinerea*. Compared with control treatment (Mock), exogenous Ile application reduced the lesion size on lettuce leaves by 18%, on white rose by 11%, on red rose by 18%, and on strawberry by 14% (Figures 5A,B). These results demonstrated that the application of Ile moderately enhanced plant resistance to *B. cinerea* in horticultural plant species, suggesting a valuable approach to control *B. cinerea* in agriculture.

## Discussion

*Botrytis cinerea* causes gray mold disease, which leads to severe economic losses (Glazebrook, 2005). The risks of fungicide resistance and pesticide residues on fresh fruits and crops are the main problems for crop protection (AbuQamar et al., 2017). It is necessary to identify safer methods to protect crops from *B. cinerea* infection. This study identified Ile as an enhancer for plant resistance to the necrotrophic pathogen *B. cinerea* in *Arabidopsis* and other horticultural plants, such as lettuces, roses, and strawberries (Figures 1–3, 5). Many kinds of signals are involved in the defense against *B. cinerea*, including JAs, ethylene (ET), SA, abscisic acid (ABA), reactive oxygen species (ROS), and NO (Thomma et al., 1998; Audenaert et al., 2002; Adie et al., 2007; Abuqamar et al., 2008; L’Haridon et al., 2011; Beneloujaephajri et al., 2013; Song et al., 2014; AbuQamar et al., 2017). Among them, JA signaling is well known as one of the most important signals in the regulation of the defense against *B. cinerea* (Abuqamar et al., 2008; Song et al., 2013, 2014; AbuQamar et al., 2017). Moreover, Ile is involved in the biosynthesis of the bioactive JA molecule: JA-Ile (Staswick and Tiryaki, 2004; Kang et al., 2006; Yan et al., 2016). Using *jar1-1* (defect in JA-Ile biosynthesis) and *coi1-1* (defect in JA-Ile perception) mutants, we found that application of Ile enhanced plant resistance against *B. cinerea* in WT but not in *jar1-1* and *coi1-1* mutants (Figure 3). These findings demonstrate that Ile acts through JA signaling depending on COI1 and JAR1 to trigger plant defense against *B. cinerea* infection.

Previous study showed that ^13^C-labeled JA-Ile was greatly synthesized in the [^13^C_6_]Ile-treated leaves accompanied with wounding treatment, which was dramatically higher than that in [^13^C_4_]Thr-treated leaves (Kang et al., 2006), suggesting that Ile could directly conjugate with JA to form JA-Ile and the conjugation capacity is limited by substrate availability. Consistently, this study showed that the application of exogenous Ile greatly improved JA-Ile concentration and transcript level of defensive gene *PDF1.2* triggered by *B. cinerea* infection (Figure 1). Furthermore, the increase of endogenous Ile level in *lib* mutant also elevated JA-Ile concentration and the transcript level of defensive gene *PDF1.2* and JA biosynthetic gene *OPR3* (Figure 2). Collectively, these findings indicate that increased Ile level could enhance plant resistance against *B. cinerea* infection *via* acting as a substrate to promote JA-Ile biosynthesis and activate JA signaling. In addition, Ile-triggered disease resistance and the acceleration of *PDF1.2* gene expression level were not observed in *jar1-1* and *coi1-1* mutants on *B. cinerea* infection (Figure 3), further suggesting that Ile modulated plant resistance to *B. cinerea* depending on JA-Ile biosynthesis and perception through JAR1 and COI1 (Figure 5C). In addition JA is synthesized from α-linolenic acid under the catalysis of a series of enzymes in plastid and peroxisome, which is then exported to the cytoplasm, where it is conjugated with Ile to form bioactive JA-Ile under the catalysis of JAR1 (Huang et al., 2017). Hence, the application of exogenous Ile may enlarge the intracellular Ile pool and provide more available Ile to act as a substrate for JA-Ile biosynthesis under the catalysis of JAR1 in the cytoplasm. In summary, our findings provide a potential strategy for crop protection *via* increasing the supply of Ile, the precursor of JA-Ile, to improve plant resistance to *B. cinerea*.

It has been reported that wounded leaves showed enhanced resistance to *B. cinerea* infection *via* producing ROS, which depends on the ABA accumulation (L’Haridon et al., 2011; Beneloujaephajri et al., 2013). JAs could also promote ROS production (Suhita et al., 2004) and interact with ABA signaling pathway (Adie et al., 2007; Brossa et al., 2011; de Ollas et al., 2015; Aleman et al., 2016). Our findings showed that mechanical wounding treatment obviously induced JA-Ile accumulation and the expression of JA biosynthetic genes (such as *OPR3*) due to 1-fold increase in the Ile levels (Figures 4A,B). Thus, it would be interesting to further investigate whether the enhanced Ile level in *lib* leaves and Ile treatment affects ROS and ABA accumulation to regulate defense responses.

*Nicotiana attenuata* mutants with less Ile contents caused by the mildly silenced *TD* gene, a homolog of the *OMR1* gene, are highly susceptible to the attack by *Manduca sexta*. Moreover, the resistance is restored *via* the application of JA-Ile or Ile (Kang et al., 2006). Together with our results that mechanical wounding enhanced JA-Ile biosynthesis and JA-responsive gene expression levels in *lib* leaves (Figures 4A,B), these findings indicate that increasing Ile level may have positive effects on plant defense against herbivore attack, but further studies need to be performed.

The application of MeJA on postharvest fruits could induce a plant defensive response and reduce the detrimental impact of pathogen infection (Yu et al., 2009; Jiang et al., 2015; Liu et al., 2016; Reyes-Diaz et al., 2016). In addition, preharvest and postharvest treatments of fruits with MeJA induced the accumulation of secondary metabolites, including anthocyanins and other antioxidant molecules, to prolong the storage period of fruits (Reyes-Diaz et al., 2016). This study reported that the increase of Ile level enhanced defensive responses to mechanical wounding or pathogen infection and MeJA-induced anthocyanin accumulation (Figures 1–4), suggesting the potential to increase secondary metabolites production and enhance plant resistance during fruit storage and transportation.

Chemical fungicide treatment is the most effective method to control *B. cinere*a, but repeated applications of fungicides increased the risk of fungicide resistance (Kretschmer et al., 2009; Rupp et al., 2016). Foliar fertilization is advantageous because it is applied at a relatively low concentration, with high efficiency in a uniform distribution, and plants respond to the application quickly (Kumar et al., 2016; Vishekaii et al., 2019). We revealed that Ile application enhanced plant resistance to *B. cinerea* in many species, including lettuce, rose, and strawberry (Figures 5A,B), which supports the potential to utilize water-soluble fertilizer containing Ile as a new efficient and safe strategy for *B. cinerea* control.

## Materials and Methods

### Plant Materials and Growth Conditions

*Arabidopsis thaliana* mutants *lib* (Yu et al., 2013), *coi1-1* (Xie et al., 1998), and *jar1-1* (Staswick et al., 2002) were described previously. In all experiments, the Columbia-0 (Col-0) was used as WT plants. Meanwhile, *coi1-1* homozygous seedlings were screened out *via* supplying JA in MS medium (Xie et al., 1998). *Arabidopsis* seeds were sterilized with 20% bleach, plated on Murashige and Skoog medium (MS; Sigma-Aldrich, United States), cooled at 4°C for 2 days, and then grown in a growth room under a 16-h light/8-h dark (21–23°C) photoperiod.

### Application of Exogenous Amino Acids

L-Ala (A7469, Sigma-Aldrich, United States), L-Leu (L8912, Sigma-Aldrich, United States), L-Ile (I2752, Sigma-Aldrich, United States), L-Met (M5308, Sigma-Aldrich, United States), L-Phe (V900489, VETEC, United States), L-Pro (V900338, VETEC, United States), L-Trp (V900470, VETEC, United States), L-Val (V900465, VETEC, United States), L-Asn (900458, VETEC, United States), L-Cys (V900400, VETEC, United States), Gly (V900144, VETEC, United States), L-Gln (V900419, VETEC, United States), L-Ser (V900406, VETEC, United States), L-Thr (V900466, VETEC, United States), L-Tyr (V900426, VETEC, United States), L-Asp (V900407, VETEC, United States), L-Glu (V900408, VETEC, United States), L-Arg (V900343, VETEC, United States), L-His (V900459, VETEC, United States), and L-Lys (V9 00409, VETEC, United States) were dissolved in 0.05% (v/v) Tween 20 solution to prepare amino acid solutions at a certain concentration. Plants grown in a room under a 10-h light/14-h dark photoperiod (21–23°C) were sprayed with amino acid solution or 0.05% Tween 20 solution (Mock), and then continued to grow for another 2 days. Subsequently, the pretreated plants were inoculated with *B. cinerea* spore suspension.

### *Botrytis cinerea* Infection Assay

*Botrytis cinerea* infection assay was based on an established method (Song et al., 2014). For leaf lesion size measurement assay, the 7th–9th rosette leaves were detached from 5-week-old plants grown under a 10-h light/14-h dark photoperiod (21–23°C), placed on 1% agar plates, inoculated with 5 μl *B. cinerea* spores (1.0 × 10^6^ spores/ml) suspended in Potato Dextrose Broth (PDB) medium, and then cultured in the dark with high humidity for 2–3 days. Subsequently, infected leaves were photographed with a digital camera, and then the lesion area was measured by using Digimizer software (v3.1.2.0, Belgium, Germany).

For JA-Ile quantification, plants were grown under a 10-h light/14-h dark photoperiod (21–23°C) for 3–4 weeks, inoculated with *B. cinerea* spores suspension (1.0 × 10^6^ spores/ml) or PDB (negative control) for 24 or 48 h under high humidity conditions. Subsequently, the 7th–9th rosette leaves were harvested for JA-Ile content quantification using LC-MS/MS as previously described (Yan et al., 2016). The experiment was performed by three biological replicates.

### Quantification of Amino Acids

For quantification of amino acids, 15-day-old plants were used. Seeds were germinated on MS medium for 5 days, and then transferred to vertical MS medium (1.2% agar) plates and grown for 10 days. Leaves were harvested for the measurement of amino acids. Here, 100 mg tissue from each sample was used. Samples were ground with liquid nitrogen. Each sample was extracted overnight at –20°C with 800 μl precooled extraction buffer [MeOH:H_2_O:HCOOH is 15:4:1 (v:v:v)]. Samples were centrifuged at the highest speed for 20 min at 4°C. Supernatants were diluted 50 times with water containing 1 ng/μl lab AA mix algae extract containing ^13^C, ^15^N-labeled amino acids (Sigma-Aldrich, catalog number: 487910). Twenty amino acids including abundant and low amino acids were detected and analyzed by UHPLC–MS/MS based on an established method (Schafer et al., 2016). For the chromatographic separation, UHPLC system equipped with a Zorbax Eclipse XDB-C18 column was used with (A) 0.1% (v/v) ACN, 0.05% (v/v) HCOOH in water and (B) MeOH as the mobile phase in gradient mode with described settings (Schafer et al., 2016). Analysis was performed on an EvoQ Triple quad-MS.

### Wound Treatment

Rosette leaves of 4-week-old plants were crushed with an hemostat. Three leaves per plant were treated. Each leaf was wounded thrice across the main leaf vein, which created a wounded area of approximately 40–50%. Each sample consisted of approximately 30 leaves from 10 plants. One hour after wounding treatment, the wounded leaves and unwounded leaves (from untreated plants) were collected for the measurement of JA-Ile content and real-time PCR. JA-Ile content was measured by using LC-MS/MS as previously described (Yan et al., 2016). The experiment was performed by three biological replicates.

### MeJA Treatment

For JA-Ile content measurement, *Arabidopsis* seedlings grown in MS medium for 14 days were treated with MeJA (0, 20, and 100 μM) for 1 h and harvested for the extraction and quantification of JA-Ile. JA-Ile content was measured by using LC-MS/MS as previously described (Yan et al., 2016). For the gene expression analysis as shown in Figure 4D, the 7-day-old seedlings grown in MS medium were soaked into solvent (Mock) and 50 μM MeJA for 1 h. The experiment was performed by three biological replicates.

### Anthocyanin Measurement

For the anthocyanin accumulation assay, *Arabidopsis* seedlings were grown in MS medium supplied with 0, 10, and 20 μM MeJA for 8 days, and then harvested for anthocyanin measurement and gene expression analysis. The phenotype and anthocyanin content were measured based on a previously described method (Song et al., 2013). Briefly, 8-day-old seedlings were photographed on a stereo microscope (Nikon), weighted, and collected in 1.5 ml tubes. We added 1 ml anthocyanin extraction buffer [*N*-propanol:H_2_O:HCl is 18:81:1 (v:v:v)] to the tube, which was boiled at 100°C for 5 min to extract anthocyanin in the dark at room temperature overnight. Anthocyanin contents are presented as (A_535_-A_650_)/g FW. The experiment was performed by three biological replicates.

### Real-Time PCR

Total RNAs were extracted from the indicated materials *via* using TransZol reagent (ET101-01, TransGen Biotech, China) and reverse transcribed by using TransScript One-Step gDNA Removal and cDNA Synthesis SuperMix (AH311-03, TransGen Biotech, China). Real-time PCR analysis was performed using Power SYBR Master Mix (A25778, ABI, United States) and the ABI7500 Real-Time PCR system. *ACTIN8* was used as the internal control. The experiment was performed by three replicates. The primers used in this study are shown in Supplementary Table 2.

### Statistical Analysis

Statistically significant differences were determined by using a two-tailed Student’s *t*-test.

### Accession Numbers

*AOS* (AT5G42650), *OPR3* (AT2G06050), *JAZ1* (AT1G19180), *JAZ5* (AT1G17380), *JAZ10* (AT5G13220), *GL3* (AT5G41315), *MYB75* (AT1G56650), *OMR1* (AT3G10050), *PDF1.2* (AT5G44420), *VSP1* (AT5G24780), and *ACTIN8* (AT1G49240).

## Data Availability Statement

The original contributions presented in the study are included in the article/Supplementary Material, further inquiries can be directed to the corresponding authors.

## Author Contributions

JY, DX, and SL were designed the study. YL and SL were performed all the experiments and analyzed the data with assistance from RD, JW, and HL. YL, SL, JY, and DX wrote the manuscript. All authors contributed to the article and approved the submitted version.

## Conflict of Interest

The authors declare that the research was conducted in the absence of any commercial or financial relationships that could be construed as a potential conflict of interest.

## Publisher’s Note

All claims expressed in this article are solely those of the authors and do not necessarily represent those of their affiliated organizations, or those of the publisher, the editors and the reviewers. Any product that may be evaluated in this article, or claim that may be made by its manufacturer, is not guaranteed or endorsed by the publisher.

## Supplementary Material

Supplementary Figure 1 Transcript level of jasmonate (JA)-responsive genes on *B. cinerea* infection.

Supplementary Figure 2 Transcript level of JA-responsive genes on MeJA treatment.

Supplementary Figure 3 *lib* mutant exhibits normal aboveground growth and development.

Supplementary Table 1 Amount of free amino acids in the leaves of wild type, *lib*, and *jar1-1* seedlings.

Supplementary Table 2 Primers used for quantitative real-time PCR (qPCR).

## References

[B1] Abuqamar S. Chai M. F. Luo H. Song F. Mengiste T. (2008). Tomato protein kinase 1b mediates signaling of plant responses to necrotrophic fungi and insect herbivory. *Plant Cell* 20 1964–1983. 10.1105/tpc.108.059477 18599583 PMC2518242

[B2] AbuQamar S. Moustafa K. Tran L. S. P. (2017). Mechanisms and strategies of plant defense against *Botrytis cinerea*. *Crit. Rev. Biotechnol.* 37 262–274. 10.1080/07388551.2016.1271767 28056558

[B3] Adie B. A. Perez-Perez J. Perez-Perez M. M. Godoy M. Sanchez-Serrano J. J. Schmelz E. A. (2007). ABA is an essential signal for plant resistance to pathogens affecting JA biosynthesis and the activation of defenses in *Arabidopsis*. *Plant Cell* 19 1665–1681. 10.1105/tpc.106.048041 17513501 PMC1913739

[B4] Aghaei K. Pirbalouti A. G. Mousavi A. Badi H. N. Mehnatkesh A. (2019). Effects of foliar spraying of L-phenylalanine and application of bio-fertilizers on growth, yield, and essential oil of hyssop [*Hyssopus officinalis* l. subsp. *Angustifolius* (Bieb.)]. *Biocatal. Agric. Biotechnol.* 21:101318. 10.1016/j.bcab.2019.101318

[B5] Albinsky D. Sawada Y. Kuwahara A. Nagano M. Hirai A. Saito K. (2010). Widely targeted metabolomics and coexpression analysis as tools to identify genes involved in the side-chain elongation steps of aliphatic glucosinolate biosynthesis. *Amino Acids* 39 1067–1075. 10.1007/s00726-010-0681-5 20623150

[B6] Aleman F. Yazaki J. Lee M. Takahashi Y. Kim A. Y. Li Z. (2016). An ABA-increased interaction of the PYL6 ABA receptor with MYC2 transcription factor: a putative link of ABA and JA signaling. *Sci. Rep.* 6:28941. 10.1038/srep28941 27357749 PMC4928087

[B7] Audenaert K. De Meyer G. B. Hofte M. M. (2002). Abscisic acid determines basal susceptibility of tomato to *Botrytis cinerea* and suppresses salicylic acid-dependent signaling mechanisms. *Plant Physiol.* 128 491–501. 10.1104/pp.010605 11842153 PMC148912

[B8] Beneloujaephajri E. Costa A. L’Haridon F. Metraux J. P. Binda M. (2013). Production of reactive oxygen species and wound-induced resistance in *Arabidopsis thaliana* against *Botrytis cinerea* are preceded and depend on a burst of calcium. *BMC Plant Biol.* 13:160. 10.1186/1471-2229-13-160 24134148 PMC4016300

[B9] Binder S. (2010). Branched-chain amino acid metabolism in *Arabidopsis thaliana*. *Arabidopsis Book* 8:e0137. 10.1199/tab.0137 22303262 PMC3244963

[B10] Brossa R. López-Carbonell M. Jubany-Marí T. Alegre L. (2011). Interplay between abscisic acid and jasmonic acid and its role in water-oxidative stress in wild-type, ABA-deficient, JA-deficient, and ascorbate-deficient *Arabidopsis* Plants. *J. Plant Growth Regul.* 30 322–333. 10.1007/s00344-011-9194-z

[B11] Cao X. Yan H. Liu X. Li D. Sui M. Wu J. (2019). A detached petal disc assay and virus-induced gene silencing facilitate the study of *Botrytis cinerea* resistance in rose flowers. *Hortic. Res.* 6:136. 10.1038/s41438-019-0219-2 31814989 PMC6885046

[B12] Creelman R. A. Mullet J. E. (1995). Jasmonic acid distribution and action in plants: regulation during development and response to biotic and abiotic stress. *Proc. Natl. Acad. Sci. U.S.A.* 92 4114–4119. 10.1146/annurev.arplant.48.1.355 11607536 PMC41895

[B13] de Ollas C. Arbona V. Gomez-Cadenas A. (2015). Jasmonoyl isoleucine accumulation is needed for abscisic acid build-up in roots of *Arabidopsis* under water stress conditions. *Plant Cell Environ.* 38 2157–2170. 10.1111/pce.12536 25789569

[B14] Dean R. Van Kan J. A. Pretorius Z. A. Hammond-Kosack K. E. Di Pietro A. Spanu P. D. (2012). The top 10 fungal pathogens in molecular plant pathology. *Mol. Plant Pathol.* 13 414–430. 10.1111/j.1364-3703.2011.00783.x 22471698 PMC6638784

[B15] Elzaki M. E. A. Li Z. F. Wang J. Xu L. Liu N. Zeng R. S. (2020). Activiation of the nitric oxide cycle by citrulline and arginine restores susceptibility of resistant brown planthoppers to the insecticide imidacloprid. *J. Hazard Mater.* 396:122755. 10.1016/j.jhazmat.2020.122755 32361135

[B16] Farmer E. E. Almeras E. Krishnamurthy V. (2003). Jasmonates and related oxylipins in plant responses to pathogenesis and herbivory. *Curr. Opin. Plant Biol.* 6 372–378. 10.1016/S1369-5266(03)00045-1 12873533

[B17] Fonseca S. Chini A. Hamberg M. Adie B. Porzel A. Kramell R. (2009). (+)-7-*iso*-Jasmonoyl-_L_-isoleucine is the endogenous bioactive jasmonate. *Nat. Chem. Biol.* 5 344–350. 10.1038/nchembio.161 19349968

[B18] Glazebrook J. (2005). Contrasting mechanisms of defense against biotrophic and necrotrophic pathogens. *Annu. Rev. Phytopathol.* 43 205–227. 10.1146/annurev.phyto.43.040204.135923 16078883

[B19] Halkier B. A. Gershenzon J. (2006). Biology and biochemistry of glucosinolates. *Annu. Rev. Plant Biol.* 57 303–333. 10.1146/annurev.arplant.57.032905.105228 16669764

[B20] Huang H. Liu B. Liu L. Song S. (2017). Jasmonate action in plant growth and development. *J. Exp. Bot.* 68 1349–1359. 10.1093/jxb/erw495 28158849

[B21] Jiang L. Jin P. Wang L. Yu X. Wang H. Zheng Y. (2015). Methyl jasmonate primes defense responses against *Botrytis cinerea* and reduces disease development in harvested table grapes. *Sci. Hortic.* 192 218–223. 10.1016/j.scienta.2015.06.015

[B22] Kadotani N. Akagi A. Takatsuji H. Miwa T. Igarashi D. (2016). Exogenous proteinogenic amino acids induce systemic resistance in rice. *BMC Plant Biol.* 16:60. 10.1186/s12870-016-0748-x 26940322 PMC4778346

[B23] Kang J. H. Wang L. Giri A. Baldwin I. T. (2006). Silencing threonine deaminase and JAR4 in *Nicotiana attenuata* impairs jasmonic acid-isoleucine-mediated defenses against *Manduca sexta*. *Plant Cell* 18 3303–3320. 10.1105/tpc.106.041103 17085687 PMC1693959

[B24] Kretschmer M. Leroch M. Mosbach A. Walker A. S. Fillinger S. Mernke D. (2009). Fungicide-driven evolution and molecular basis of multidrug resistance in field populations of the grey mould fungus *Botrytis cinerea*. *PLoS Pathog.* 5:e1000696. 10.1371/journal.ppat.1000696 20019793 PMC2785876

[B25] Kumar J. Kumar R. Rai R. Mishra D. S. Singh S. K. Nimbolkar P. K. (2016). Influence of foliar application of mineral nutrients at different growth stages of guava. *J. Plant Nutr.* 40 656–661. 10.1080/01904167.2016.1246568

[B26] L’Haridon F. Besson-Bard A. Binda M. Serrano M. Abou-Mansour E. Balet F. (2011). A permeable cuticle is associated with the release of reactive oxygen species and induction of innate immunity. *PLoS Pathog.* 7:e1002148. 10.1371/journal.ppat.1002148 21829351 PMC3145797

[B27] Liu Y. Yang X. Zhu S. Wang Y. (2016). Postharvest application of MeJA and NO reduced chilling injury in cucumber (*Cucumis sativus*) through inhibition of H_2_O_2_ accumulation. *Postharvest Biol. Technol.* 119 77–83. 10.1016/j.postharvbio.2016.04.003

[B28] Petrasch S. Knapp S. J. Van Kan J. A. L. Blanco-Ulate B. (2019). Grey mould of strawberry, a devastating disease caused by the ubiquitous necrotrophic fungal pathogen *Botrytis cinerea*. *Mol. Plant Pathol.* 20 877–892. 10.1111/mpp.12794 30945788 PMC6637890

[B29] Qi T. Song S. Ren Q. Wu D. Huang H. Chen Y. (2011). The Jasmonate-ZIM-domain proteins interact with the WD-Repeat/bHLH/MYB complexes to regulate Jasmonate-mediated anthocyanin accumulation and trichome initiation in *Arabidopsis thaliana*. *Plant Cell* 23 1795–1814. 10.1105/tpc.111.083261 21551388 PMC3123955

[B30] Qi W. Chen X. Fang P. Shi S. Li J. Liu X. (2018). Genomic and transcriptomic sequencing of *Rosa hybrida* provides microsatellite markers for breeding, flower trait improvement and taxonomy studies. *BMC Plant Biol.* 18:119. 10.1186/s12870-018-1322-5 29907083 PMC6003205

[B31] Rahmani Samani M. Ghasemi Pirbalouti A. Moattar F. Golparvar A. R. (2019). L-Phenylalanine and bio-fertilizers interaction effects on growth, yield and chemical compositions and content of essential oil from the sage (*Salvia officinalis* L.) leaves. *Ind. Crops Prod.* 137 1–8. 10.1016/j.indcrop.2019.05.019

[B32] Reyes-Diaz M. Lobos T. Cardemil L. Nunes-Nesi A. Retamales J. Jaakola L. (2016). Methyl jasmonate: an alternative for improving the quality and health properties of fresh fruits. *Molecules* 21:567. 10.3390/molecules21060567 27258240 PMC6273056

[B33] Rupp S. Weber R. W. Rieger D. Detzel P. Hahn M. (2016). Spread of *Botrytis cinerea* strains with multiple fungicide resistance in German horticulture. *Front. Microbiol.* 7:2075. 10.3389/fmicb.2016.02075 28096799 PMC5206850

[B34] Schafer M. Brutting C. Baldwin I. T. Kallenbach M. (2016). High-throughput quantification of more than 100 primary- and secondary-metabolites, and phytohormones by a single solid-phase extraction based sample preparation with analysis by UHPLC-HESI-MS/MS. *Plant Methods* 12:30. 10.1186/s13007-016-0130-x 27239220 PMC4882772

[B35] Seo J. S. Joo J. Kim M. J. Kim Y. K. Nahm B. H. Song S. I. (2011). OsbHLH148, a basic helix-loop-helix protein, interacts with OsJAZ proteins in a jasmonate signaling pathway leading to drought tolerance in rice. *Plant J.* 65 907–921. 10.1111/j.1365-313X.2010.04477.x 21332845

[B36] Shim C. K. Kim M. J. Kim Y. K. Jee H. J. (2014). Evaluation of lettuce germplasm resistance to gray mold disease for organic cultivations. *Plant Pathol. J.* 30 90–95. 10.5423/PPJ.NT.07.2013.0064 25288990 PMC4174840

[B37] Song S. Huang H. Gao H. Wang J. Wu D. Liu X. (2014). Interaction between MYC2 and ETHYLENE INSENSITIVE3 modulates antagonism between jasmonate and ethylene signaling in *Arabidopsis*. *Plant Cell* 26 263–279. 10.1105/tpc.113.120394 24399301 PMC3963574

[B38] Song S. Qi T. Fan M. Zhang X. Gao H. Huang H. (2013). The bHLH subgroup IIId factors negatively regulate jasmonate-mediated plant defense and development. *PLoS Genet.* 9:e1003653. 10.1371/journal.pgen.1003653 23935516 PMC3723532

[B39] Staswick P. E. Tiryaki I. (2004). The oxylipin signal jasmonic acid is activated by an enzyme that conjugates it to isoleucine in *Arabidopsis*. *Plant Cell* 16 2117–2127. 10.1105/tpc.104.023549 15258265 PMC519202

[B40] Staswick P. E. Tiryaki I. Rowe M. L. (2002). Jasmonate response locus JAR1 and several related *Arabidopsis* genes encode enzymes of the firefly luciferase superfamily that show activity on jasmonic, salicylic, and indole-3-acetic acids in an assay for adenylation. *Plant Cell* 14 1405–1415. 10.1105/tpc.000885 12084835 PMC150788

[B41] Suhita D. Raghavendra A. S. Kwak J. M. Vavasseur A. (2004). Cytoplasmic alkalization precedes reactive oxygen species production during methyl jasmonate- and abscisic acid-induced stomatal closure. *Plant Physiol.* 134 1536–1545. 10.1104/pp.103.032250 15064385 PMC419829

[B42] Suza W. P. Staswick P. E. (2008). The role of JAR1 in Jasmonoyl-_L_-isoleucine production during *Arabidopsis* wound response. *Planta* 227 1221–1232. 10.1007/s00425-008-0694-4 18247047

[B43] Tamogami S. Rakwal R. Agrawal G. K. (2008). Interplant communication: airborne methyl jasmonate is essentially converted into JA and JA-Ile activating jasmonate signaling pathway and VOCs emission. *Biochem. Biophys. Res. Commun.* 376 723–727. 10.1016/j.bbrc.2008.09.069 18812165

[B44] Thaler J. S. Owen B. Higgins V. J. (2004). The role of the jasmonate response in plant susceptibility to diverse pathogens with a range of lifestyles. *Plant Physiol.* 135 530–538. 10.1104/pp.104.041566 15133157 PMC429405

[B45] Thines B. Katsir L. Melotto M. Niu Y. Mandaokar A. Liu G. (2007). JAZ repressor proteins are targets of the SCF^COI1^ complex during jasmonate signalling. *Nature* 448 661–665. 10.1038/nature05960 17637677

[B46] Thomma B. P. Eggermont K. Penninckx I. A. Mauch-Mani B. Vogelsang R. Cammue B. P. (1998). Separate jasmonate-dependent and salicylate-dependent defense-response pathways in *Arabidopsis* are essential for resistance to distinct microbial pathogens. *Proc. Natl. Acad. Sci. U.S.A.* 95 15107–15111. 10.1073/pnas.95.25.15107 9844023 PMC24583

[B47] Vishekaii Z. R. Soleimani A. Fallahi E. Ghasemnezhad M. Hasani A. (2019). The impact of foliar application of boron nano-chelated fertilizer and boric acid on fruit yield, oil content, and quality attributes in olive (*Olea europaea* L.). *Sci. Hortic.* 257:108689. 10.1016/j.scienta.2019.108689

[B48] Wang J. Liu Z. Wang Y. Cheng W. Mou H. (2014). Production of a water-soluble fertilizer containing amino acids by solid-state fermentation of soybean meal and evaluation of its efficacy on the rapeseed growth. *J. Biotechnol.* 187 34–42. 10.1016/j.jbiotec.2014.07.015 25062659

[B49] Wasternack C. Hause B. (2013). Jasmonates: biosynthesis, perception, signal transduction and action in plant stress response, growth and development. An update to the 2007 review in Annals of Botany. *Ann. Bot.* 111 1021–1058. 10.1093/aob/mct067 23558912 PMC3662512

[B50] Wathugala D. L. Hemsley P. A. Moffat C. S. Cremelie P. Knight M. R. Knight H. (2012). The Mediator subunit SFR6/MED16 controls defence gene expression mediated by salicylic acid and jasmonate responsive pathways. *New Phytol.* 195 217–230. 10.1111/j.1469-8137.2012.04138.x 22494141

[B51] Williamson B. Tudzynski B. Tudzynski P. Van Kan J. A. (2007). *Botrytis cinerea*: the cause of grey mould disease. *Mol. Plant Pathol.* 8 561–580. 10.1111/j.1364-3703.2007.00417.x 20507522

[B52] Windram O. Madhou P. McHattie S. Hill C. Hickman R. Cooke E. (2012). *Arabidopsis* defense against *Botrytis cinerea*: chronology and regulation deciphered by high-resolution temporal transcriptomic analysis. *Plant Cell* 24 3530–3557. 10.1105/tpc.112.102046 23023172 PMC3480286

[B53] Xie D. X. Feys B. F. James S. Nieto-Rostro M. Turner J. G. (1998). COI1: an *Arabidopsis* gene required for jasmonate-regulated defense and fertility. *Science* 280 1091–1094. 10.1126/science.280.5366.1091 9582125

[B54] Xu L. Liu F. Lechner E. Genschik P. Crosby W. L. Ma H. (2002). The SCF^COI1^ ubiquitin-ligase complexes are required for jasmonate response in *Arabidopsis*. *Plant Cell* 14 1919–1935. 10.1105/tpc.003368 12172031 PMC151474

[B55] Yan C. Fan M. Yang M. Zhao J. Zhang W. Su Y. (2018). Injury activates Ca^2+^/Calmodulin-dependent phosphorylation of JAV1-JAZ8-WRKY51 complex for jasmonate biosynthesis. *Mol. Cell* 70 136–149. 10.1016/j.molcel.2018.03.013 29625034

[B56] Yan J. Li H. Li S. Yao R. Deng H. Xie Q. (2013). The *Arabidopsis* F-box protein CORONATINE INSENSITIVE1 is stabilized by SCF^*COI1*^ and degraded via the 26S proteasome pathway. *Plant Cell* 25 486–498. 10.1105/tpc.112.105486 23386265 PMC3608773

[B57] Yan J. Li S. Gu M. Yao R. Li Y. Chen J. (2016). Endogenous bioactive jasmonate is composed of a set of (+)-7-*iso*-JA-amino acid conjugates. *Plant Physiol.* 172 2154–2164. 10.1104/pp.16.00906 27756820 PMC5129707

[B58] Yan J. Zhang C. Gu M. Bai Z. Zhang W. Qi T. (2009). The *Arabidopsis* CORONATINE INSENSITIVE1 protein is a jasmonate receptor. *Plant Cell* 21 2220–2236. 10.1105/tpc.109.065730 19717617 PMC2751961

[B59] Yu H. Zhang F. Wang G. Liu Y. Liu D. (2013). Partial deficiency of isoleucine impairs root development and alters transcript levels of the genes involved in branched-chain amino acid and glucosinolate metabolism in *Arabidopsis*. *J. Exp. Bot.* 64 599–612. 10.1093/jxb/ers352 23230023 PMC3542050

[B60] Yu M. Shen L. Fan B. Zhao D. Zheng Y. Sheng J. (2009). The effect of MeJA on ethylene biosynthesis and induced disease resistance to *Botrytis cinerea* in tomato. *Postharvest Biol. Technol.* 54 153–158. 10.1016/j.postharvbio.2009.07.001

