# Supplementary material for: Isoleucine Enhances Plant Resistance Against Botrytis cinerea via Jasmonate Signaling Pathway
Source: Front Plant Sci. 2021 Aug 19;12:628328. doi: 10.3389/fpls.2021.628328 (PMC8416682; doi:10.3389/fpls.2021.628328)
Supplement: Supplementary Figure 1 — Transcript level of jasmonate (JA)-responsive genes on B. cinerea infection. [file Presentation_1.pptx]

## Slide 1
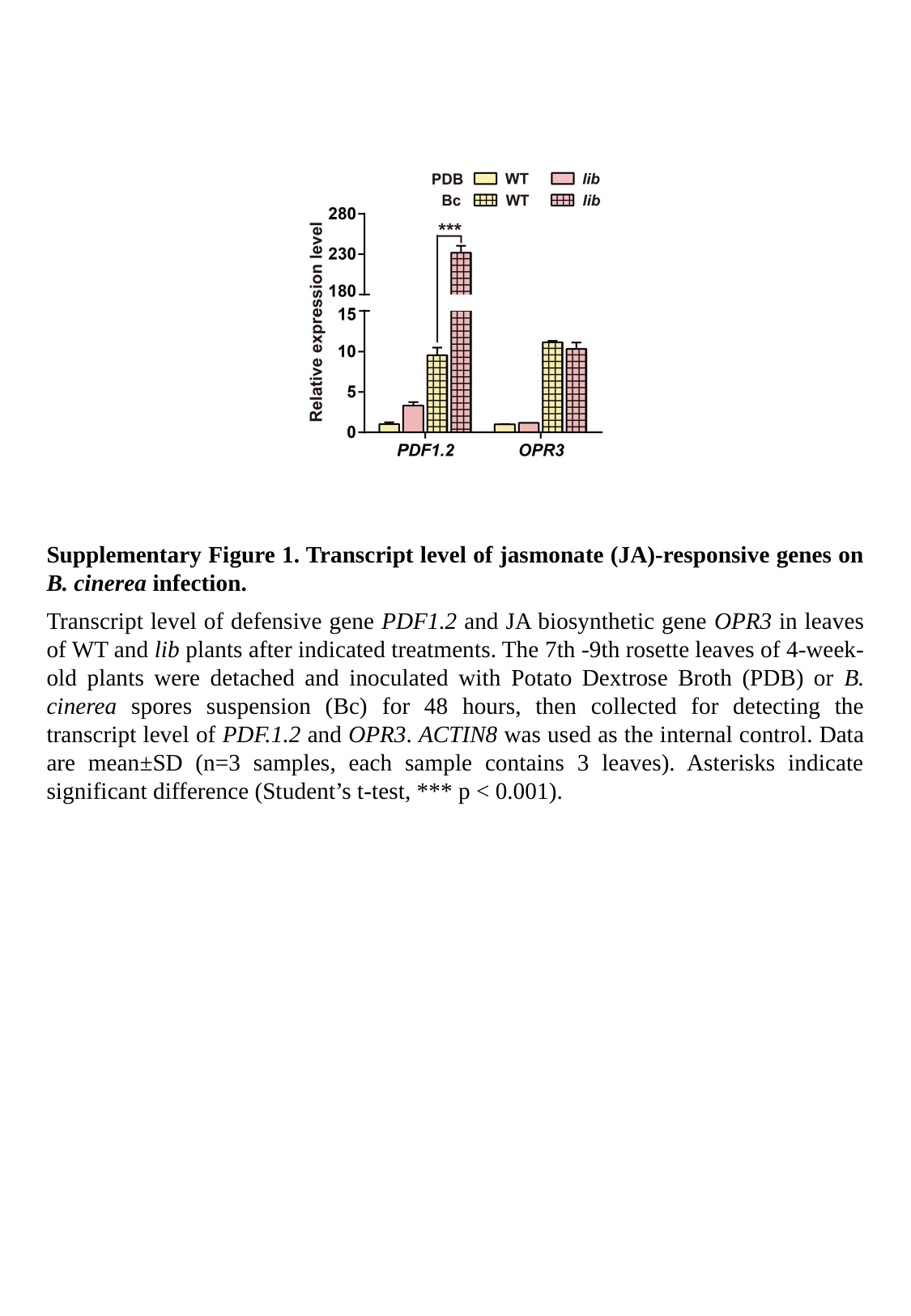

Supplementary Figure 1. Transcript level of jasmonate (JA)-responsive genes on B. cinerea infection.
Transcript level of defensive gene PDF1.2 and JA biosynthetic gene OPR3 in leaves of WT and lib plants after indicated treatments. The 7th -9th rosette leaves of 4-week-old plants were detached and inoculated with Potato Dextrose Broth (PDB) or B. cinerea spores suspension (Bc) for 48 hours, then collected for detecting the transcript level of PDF.1.2 and OPR3. ACTIN8 was used as the internal control. Data are mean±SD (n=3 samples, each sample contains 3 leaves). Asterisks indicate significant difference (Student’s t-test, *** p < 0.001).
