# Supplementary material for: Isoleucine Enhances Plant Resistance Against Botrytis cinerea via Jasmonate Signaling Pathway
Source: Front Plant Sci. 2021 Aug 19;12:628328. doi: 10.3389/fpls.2021.628328 (PMC8416682; doi:10.3389/fpls.2021.628328)
Supplement: Supplementary Figure 2 — Transcript level of JA-responsive genes on MeJA treatment. [file Presentation_2.pptx]

## Slide 1
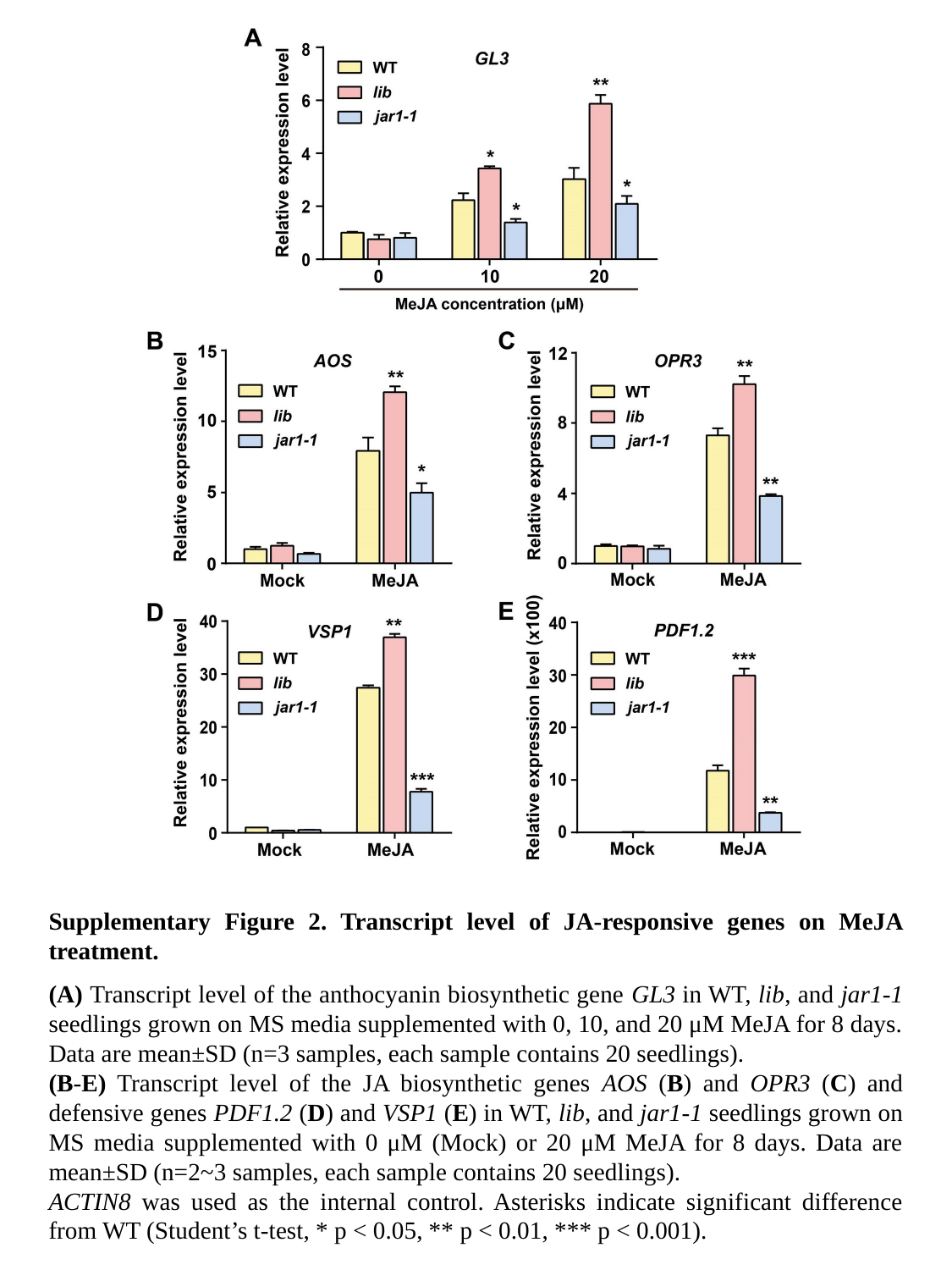

Supplementary Figure 2. Transcript level of JA-responsive genes on MeJA treatment.
(A) Transcript level of the anthocyanin biosynthetic gene GL3 in WT, lib, and jar1-1 seedlings grown on MS media supplemented with 0, 10, and 20 μM MeJA for 8 days. Data are mean±SD (n=3 samples, each sample contains 20 seedlings).
(B-E) Transcript level of the JA biosynthetic genes AOS (B) and OPR3 (C) and defensive genes PDF1.2 (D) and VSP1 (E) in WT, lib, and jar1-1 seedlings grown on MS media supplemented with 0 μM (Mock) or 20 μM MeJA for 8 days. Data are mean±SD (n=2~3 samples, each sample contains 20 seedlings).
ACTIN8 was used as the internal control. Asterisks indicate significant difference from WT (Student’s t-test, * p < 0.05, ** p < 0.01, *** p < 0.001).
