# Supplementary material for: Isoleucine Enhances Plant Resistance Against Botrytis cinerea via Jasmonate Signaling Pathway
Source: Front Plant Sci. 2021 Aug 19;12:628328. doi: 10.3389/fpls.2021.628328 (PMC8416682; doi:10.3389/fpls.2021.628328)
Supplement: Supplementary Figure 3 — lib mutant exhibits normal aboveground growth and development. [file Presentation_3.pptx]

## Slide 1
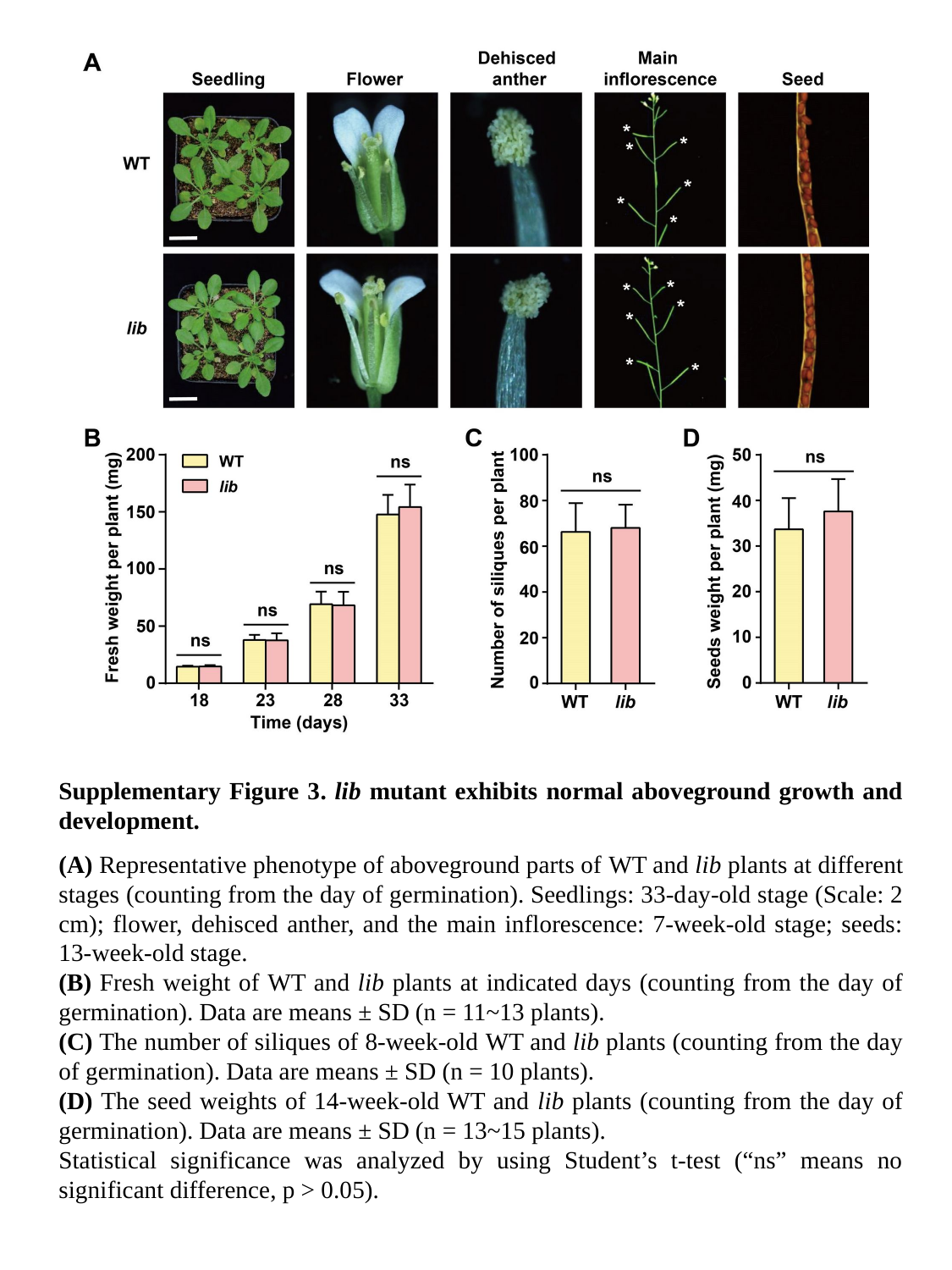

Supplementary Figure 3. lib mutant exhibits normal aboveground growth and development.
(A) Representative phenotype of aboveground parts of WT and lib plants at different stages (counting from the day of germination). Seedlings: 33-day-old stage (Scale: 2 cm); flower, dehisced anther, and the main inflorescence: 7-week-old stage; seeds: 13-week-old stage.
(B) Fresh weight of WT and lib plants at indicated days (counting from the day of germination). Data are means ± SD (n = 11~13 plants).
(C) The number of siliques of 8-week-old WT and lib plants (counting from the day of germination). Data are means ± SD (n = 10 plants).
(D) The seed weights of 14-week-old WT and lib plants (counting from the day of germination). Data are means ± SD (n = 13~15 plants).
Statistical significance was analyzed by using Student’s t-test (“ns” means no significant difference, p > 0.05).
