# Supplementary material for: Isoleucine Enhances Plant Resistance Against Botrytis cinerea via Jasmonate Signaling Pathway
Source: Front Plant Sci. 2021 Aug 19;12:628328. doi: 10.3389/fpls.2021.628328 (PMC8416682; doi:10.3389/fpls.2021.628328)
Supplement: Supplementary Table 1 — Amount of free amino acids in the leaves of wild type, lib, and jar1-1 seedlings. [file Table_1.docx]

**Supplementary Table 1.**

The amount of free amino acids (nmol/g FW) in the leaves of wild type (WT), *lib*, and *jar1-1* seedlings.

| Amino acids | WT | *lib* | *jar1-1* |
| --- | --- | --- | --- |
| Ala | 7.7 ± 0.9 | 7.5 ± 1.4 | 8.2 ± 1.6 |
| Leu | 1.4 ± 0.3 | 1.5 ± 0.2 | 1.4 ± 0.1 |
| Ile | 3.8 ± 0.6 | 8.5 ± 1.2 b | 3.3 ± 1.1 |
| Met | 38.0 ± 4.5 | 39.8 ± 4.8 | 38.5 ± 4.0 |
| Phe | 1.74 ± 0.23 | 1.72 ± 0.17 | 1.58 ± 0.10 |
| Pro | 325.1 ± 42.0 | 312.4 ± 41.8 | 373.5 ± 57.3 b |
| Trp | 4.2 ± 0.7 | 4.1 ± 0.8 | 4.7 ± 0.8 |
| Val | 8.5 ± 0.7 | 8.4 ± 0.8 | 8.3 ± 0.8 |
| Asn | 14.2 ± 1.3 | 12.6 ± 1.3 a | 18.2 ± 1.4 b |
| Cys | 19.1 ± 3.1 | 19.4 ± 5.2 | 20.7 ± 4.2 |
| Gly | 7.1 ± 1.8 | 10.3 ± 2.4 b | 3.7 ± 0.6 a |
| Gln | 103.5 ± 12.8 | 111.6 ± 12.8 | 170.7 ± 23.3 b |
| Ser | 190.8 ± 17.0 | 135.8 ± 12.7 a | 197.6 ± 20.6 |
| Thr | 24.7 ± 3.3 | 22.7 ± 2.1 | 25.4 ± 2.8 |
| Tyr | 6.2 ± 1.0 | 4.7 ± 0.6 a | 5.5 ± 1.4 |
| Asp | 23.0 ± 1.2 | 23.0 ± 1.4 | 25.2 ± 3.1 |
| Glu | 65.1 ± 6.6 | 51.0 ±5.9 a | 72.5 ± 10.0 b |
| Arg | 50.4 ± 6.8 | 54.8 ± 9.2 | 65.4 ± 7.6 b |
| His | 49.8 ± 3.7 | 42.8 ± 3.1 a | 64.2 ± 5.9 b |
| Lys | 210.8 ± 35.1 | 194.0 ± 20.7 | 315.7 ± 30.9 b |

Data are Mean ± SD (n=7~10 samples). Statistical significances were analyzed by using Student’s t-test.

“a” means that the values significantly lower than WT (p < 0.05).

“b” means that the values significantly higher than WT (p < 0.05).
