# Supplementary material for: Isoleucine Enhances Plant Resistance Against Botrytis cinerea via Jasmonate Signaling Pathway
Source: Front Plant Sci. 2021 Aug 19;12:628328. doi: 10.3389/fpls.2021.628328 (PMC8416682; doi:10.3389/fpls.2021.628328)
Supplement: Supplementary Table 2 — Primers used for quantitative real-time PCR (qPCR). [file Table_2.docx]

**Supplementary Table 2.** Primers used for quantitative real-time PCR (qPCR).

| qPCR Primers | Sequence 5’ to 3’ |
| --- | --- |
| *ACTIN8* -F | TCAGCACTTTCCAGCAGATG |
| *ACTIN8* -R | CTGTGGACAATGCCTGGAC |
| *AOS* -F | CACCGGCGTTAGTCAAATCT |
| *AOS* -R | CCGGCGGATTCTAAGAAAA |
| *OPR3* -F | AAGATTCGATCTCTCTCATCGAGT |
| *OPR3* -R | GGAGTGGTCCGTTGAGCA |
| *JAZ1* -F | GAGCAAAGGCACCGCTAATA |
| *JAZ1* -R | TGCGATAGTAGCGATGTTGC |
| *JAZ5* -F | AAAGATGTTGCTGACCTCAG TG |
| *JAZ5* -R | CCCTCCGAAGAATATGGTCA |
| *JAZ10* -F | CGCTCCTAAGCCTAAGTTCCA |
| *JAZ10* -R | TCGAAATCGCACCTTGAATA |
| *MYB75* -F | AAATGGCACCAAGTTCCTGT |
| *MYB75* -R | TCAGAGCTAAGTTTTCCTCTCTTGAT |
| *GL3* -F | AGTGTTTAGCCGTTCTCTTC TAGC |
| *GL3* -R | TGTCTTCCGTAATATGTTCTGTGG |
| *PDF1.2* -F | GTTCTCTTTGCTGCTTTCGAC |
| *PDF1.2* -R | GCAAACCCCTGACCATGT |
| *VSP1* -F | ACGTCCAGTCTTCGGCATCC |
| *VSP1* -R | TAGTTGATGGACAGTCCCTC |
| *OMR1* -F | gaaagatcacctgcgttacttga |
| *OMR1* -R | ggcatagaacctcgtctcca |
